# Involvement of p38 Activation and Mitochondria in Death of Human Leukemia Cells Induced by an Agonistic Human Monoclonal Antibody Fab Specific to TRAIL Receptor 1

**DOI:** 10.3390/ijms20081967

**Published:** 2019-04-22

**Authors:** You-Ri Lee, Eunjoo Hwang, Young-Ju Jang

**Affiliations:** Department of Microbiology, Ajou University School of Medicine, Suwon 16499, Korea; hit8311@naver.com (Y.-R.L.); eunjoo267@hanmail.net (E.H.)

**Keywords:** agonistic human antibody, Fab, TRAIL, TRAIL-resistance, cancer cells, TRAIL receptor 1, DR4, p38, mitochondria

## Abstract

The tumor necrosis factor-related apoptosis-inducing ligand (TRAIL) induces cancer cell death with minimal damage to normal cells; however, some cancer cells are resistant to TRAIL. TRAIL resistance may be overcome by agonistic antibodies to TRAIL receptors. In this study, we report the toxic effects of a novel recombinant agonistic human anti–TRAIL receptor 1 (DR4) monoclonal antibody Fab fragment, DR4-4, on various TRAIL-resistant and -sensitive cancer cell lines. The mechanisms of DR4-4 Fab–induced cell death in a human T cell leukemia cell line (Jurkat) were investigated using cell viability testing, immunoblotting, immunoassays, flow cytometry, and morphological observation. DR4-4 Fab–induced caspase-independent necrosis was observed to occur in Jurkat cells in association with p38 mitogen-activated protein kinase activation, cellular FLICE (FADD-like IL-1β-converting enzyme)-inhibitory protein degradation, decreased mitochondrial membrane potential, and increased mitochondrial reactive oxygen species production. Increased cytotoxic effects of DR4-4 Fab were observed in combination with TRAIL or γ-irradiation. Our results indicate that the novel DR4-4 Fab might overcome TRAIL-resistance and induce death in leukemia cells via cellular mechanisms different from those activated by TRAIL. DR4-4 Fab may have application as a potential therapeutic antibody fragment in single or combination therapy for cancer.

## 1. Introduction

Tumor necrosis factor (TNF)–related apoptosis-inducing ligand (TRAIL), also known as Apo2 ligand, exists in either a membrane-bound or soluble form. TRAIL has strong antitumor activity, with minimal toxicity to most normal cells [1,2,3]. The selective toxicity of TRAIL to tumor cells is mainly due to the presence of TRAIL receptors (TRAIL receptor 1/death receptor (DR) 4 and TRAIL receptor 2/DR5) containing death domains on the membrane of tumor cells [4,5,6,7]. The efficacy of soluble TRAIL (sTRAIL) as a single agent or in combination with other anticancer agents has been demonstrated [8,9]. However, sTRAIL can bind both TRAIL DRs and decoy receptors (DcR1 and DcR2), which lack death domains and are expressed in normal cells at high levels, leading to the inhibition of cell death [10]. Several intracellular signaling molecules, including cellular FLICE (FADD-like IL-1β-converting enzyme)-inhibitory protein (c-FLIP), inhibitor of apoptosis proteins, p38 mitogen-activated protein kinase (p38 MAPK), and nuclear factor (NF)-κB, have been implicated in the regulation of TRAIL resistance in tumor cells [11,12,13,14,15]. The binding of sTRAIL to DRs can induce various types of cell death in tumor cells, including apoptosis [16], autophagy [17,18], and necrosis [19,20]. The effective application of TRAIL as a cancer therapeutic agent will ultimately depend on its rational combination with drugs overcoming TRAIL resistance. 

Agonistic human or mouse monoclonal antibodies (mAbs) that specifically activate DR4 or DR5 without triggering DcR1 and DcR2 have been demonstrated as potential candidates for cancer therapy [2,21,22,23,24,25,26,27,28]. Different mAbs to DR4 and DR5 can exert their cytotoxic activities via various mechanisms. Some mAbs against DRs induce a caspase-dependent mechanism mimicking that of sTRAIL [29,30,31]. However, a mouse anti-DR5 mAb, AD5-10, induced both caspase-dependent and -independent cell death in Jurkat cells [23]. Zaptuzumab, a humanized version of AD5-10, induced caspase-dependent apoptosis and autophagic cell death [31]. Human agonistic mAbs HGS-ETR1 (anti-DR4) and HGS-ETR2 (anti-DR5) killed myeloma and lymphoma cells through caspase activation and cleavage of myeloid cell leukemia-1L [29]. Mapatumumab, a human agonistic anti-DR4 mAb, induced mitochondria-independent apoptosis [30]. A human mAb against DR5, lexatumumab, showed some clinical effectiveness for pediatric solid tumors, where, in combination with radiation therapy, it enhanced antitumor activity [32]. Many human and humanized agonists of DR4 and DR5 including the abovementioned antibodies (Abs) are currently being studied in preclinical or clinical trials for various cancers [28,31,32,33]. 

In this article, we characterized DR4-4, a novel recombinant human monovalent mAb Fab fragment produced against human recombinant DR4 antigen by phage display technology. DR4-4 Fab induced cell death in several TRAIL-sensitive and -resistant cancer cell lines; it did not induce death in normal cells. DR4-4 Fab induced necrosis-like cell death that was accompanied by mitochondrial reactive oxygen species (ROS) production, p38 MAPK activation, and decreased mitochondrial membrane potential (MMP) without activating caspases. Further, DR4-4 Fab enhanced cytotoxic effects in combination with TRAIL or γ-irradiation in human T cell leukemia cells. The cytotoxic mechanism of DR4-4 Fab is different from that of TRAIL, and this Fab fragment might be a potential candidate for combination therapy in lymphoma. 

## 2. Results

### 2.1. Characterization of a Human mAb Fab against DR4

**Figure 1:**
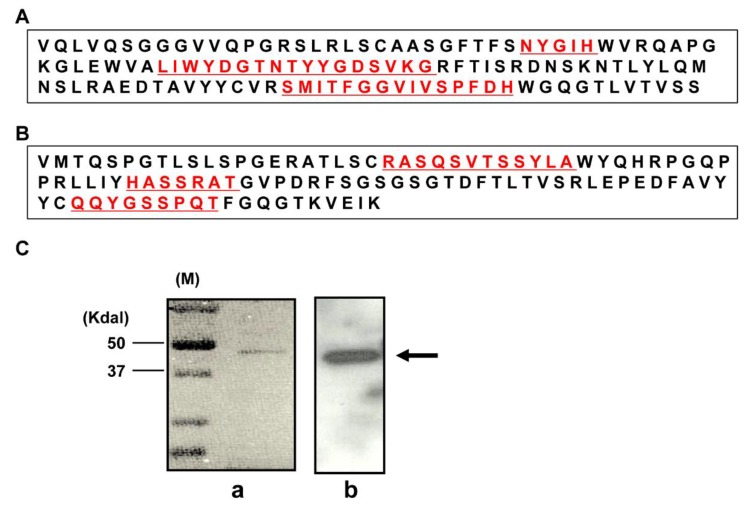
Amino acid sequences of heavy (V_H_) and light (V_L_) chains and visualization of the purified DR4-4 Fab. The amino acid sequences of the V_H_ (**A**) and V_L_ (**B**) regions of DR4-4 Fab are available from European Molecular Biology Laboratory/GenBank under accession numbers JN030159 (V_H_) and JN030158 (V_L_). The purified DR4-4 Fab (1 µg/mL for immunoblotting and 10 µg/mL for Coomassie blue staining) was visualized by sodium dodecyl sulfate-polyacrylamide gel electrophoresis (SDS-PAGE) and immunoblotting with anti–human IgG (Fab specific) monoclonal antibody (**Ca**) and Coomassie blue staining (**Cb**). DR4-4 Fab is presented by an arrow at approximately 45 kDa.

We generated a novel agonistic human recombinant Fab fragment, DR4-4, against the DR4 receptor. Sequences of the variable regions of the heavy (V_H_) and light (V_L_) chains were determined at the cDNA level. The V_H_ sequence was composed of 372 nucleotides and showed similarity to *IGHV3-33* (97.3%), *IGHD3-16* (100%), and *IGHJ4* (97.7%). The V_L_ sequence was composed of 318 nucleotides and showed similarity to *IGKV3-20* (96.1%) and *IGKJ1* (97.1%). The amino acid sequences of V_H_ and V_L_ are shown in Figure 1A,B, respectively. Three complementarity determining regions of each chain are presented in red and underlined. The expressed and purified DR4-4 Fab was visualized at an estimated size of approximately 45 kDa through immunoblotting using anti–human IgG (Fab specific) Ab (Figure 1Ca) and Coomassie blue staining (Figure 1Cb). 

**Figure 2:**
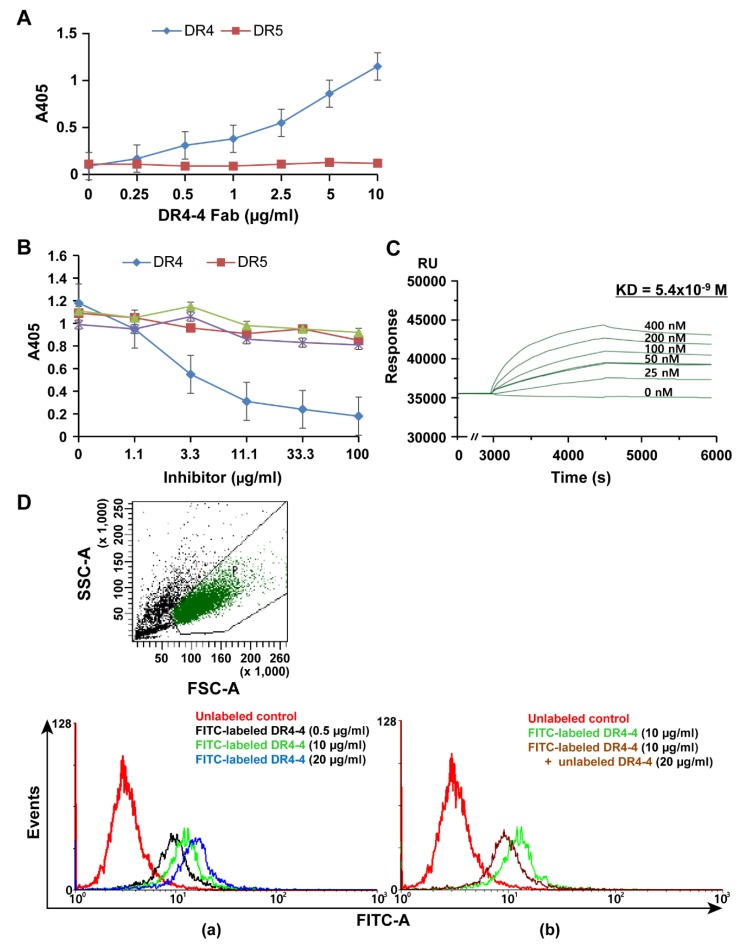
Specific binding of DR4-4 Fab to DR4 antigen. Direct-binding (**A**) and competitive (**B**) enzyme-linked immunosorbent assay (ELISA) for specific binding of DR4-4 Fab to DR4. Recombinant DR4 and DR5 were coated onto the wells of ELISA plates at 5 µg/mL, followed by incubation with DR4-4 Fab (**A**) or DR4-4 Fab preincubated with competitor, DR4 or DR5 (**B**) (data presented as mean ± standard deviation). (**C**) Binding affinity of recombinant human DR4 antigen for DR4-4 (1 µM) immobilized on a nitrile triacetic acid chip as measured by Biacore surface plasmon resonance. (**D**) Fluorescence-activated cell sorting analysis of the cellular binding of DR4-4 Fab. Cells (5 × 10^5^) were incubated with fluorescein isothiocyanate (FITC)-labeled DR4-4 Fab for 30 min at 4 °C without (**a**) or with (**b**) pretreatment with unlabeled DR4-4 Fab. Dot plot presents the profile of forward scatter (FSC)/side scatter (SSC) of control cells. P is a gate of cells which were used for the analysis. (**C**,**D**) are representative results among triplicate experiments.

A direct-binding enzyme-linked immunosorbent assay (ELISA) using recombinant human DR4 or DR5 as antigen coated onto the wells of 96-well plates was performed to demonstrate specific binding of DR4-4 Fab to DR4 (Figure 2A). At various concentrations (0.25–10 μg/mL), the purified DR4-4 Fab bound to DR4 (5 μg/mL) in a dose-dependent manner, whereas it did not bind to DR5, even at high concentrations of DR4-4 Fab. Specific binding of DR4-4 Fab to DR4 was confirmed by competitive ELISA using DRs (DR4 and DR5) and decoy receptors (DcR1 and DcR2) as competitors (Figure 2B). Preincubation of DR4-4 Fab (10 μg/mL) with DR4 at various concentrations (1.1–100 μg/mL) significantly inhibited the binding of the Fab to DR4 (5 μg/mL) coated onto the wells in a dose-dependent manner. Competition with other antigens (DR5, DcR1, and DcR2) was not remarkable, even at competitor concentrations of 100 μg/mL. Surface plasmon resonance (SPR) sensorgrams demonstrated the high binding affinity (Kd = 5.4 × 10^−9^ M) of DR4-4 Fab for DR4 (Figure 2C). 

Binding of the DR4-4 Fab to Jurkat (human T cell leukemia) cells, which express DR4 on their surface, was analyzed by flow cytometry after incubation with fluorescein isothiocyanate (FITC)-labeled DR4-4 Fab at 0.5, 10, and 20 μg/mL at 4 °C (Figure 2Da). A shift in the fluorescence signal to the right along the x-axis was observed to occur in a dose-dependent manner, indicating the cellular binding of DR4-4 Fab. Preincubation of cells with unlabeled DR4-4 Fab (20 μg/mL) at 4 °C inhibited the cellular binding of FITC-labeled DR4-4 Fab (10 μg/mL) (Figure 2Db), confirming the binding of the Fab to the surface of cells. 

### 2.2. DR4-4 Induces Cell Death in Various Cancer Cells

**Figure 3:**
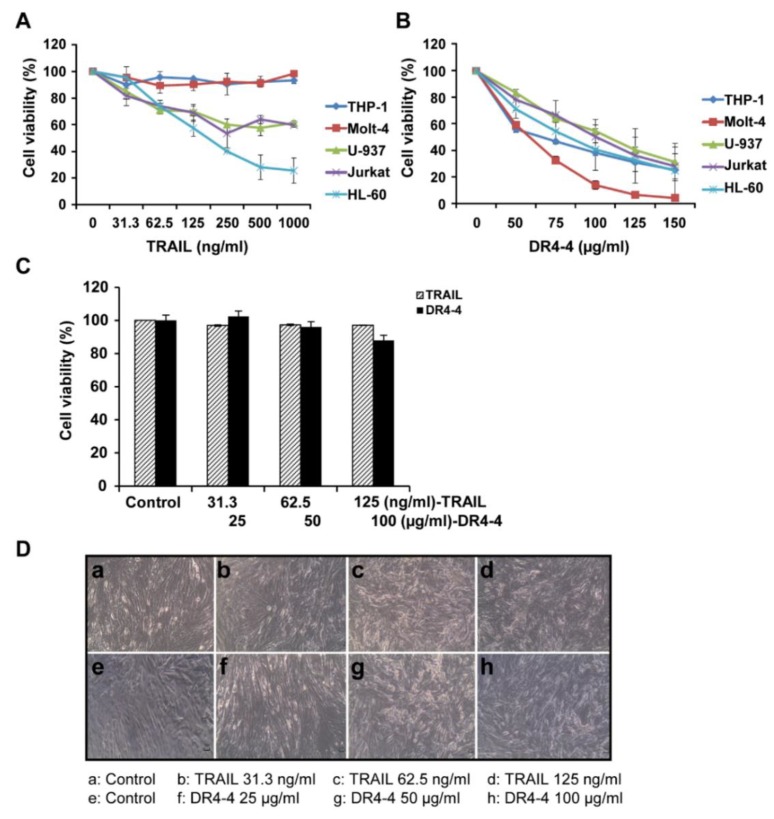
Specific cytotoxic effects of DR4-4 Fab on cancer cells. Cells (2.5 × 10^4^) were treated with the indicated concentrations of TRAIL (**A**) or DR4-4 Fab (**B**) for 48 h. (**C**) Viability (as assessed by MTT assay) of normal human fibroblasts (1 × 10^5^) treated with TRAIL or DR4-4 Fab for 48 h. (**D**) Cell morphology of normal human fibroblasts treated with TRAIL or DR4-4 Fab for 48 h was evaluated by phase contrast microscopy at 100×. Data presented as mean ± standard deviation.

The cytotoxicity of TRAIL and DR4-4 Fab at various concentrations was compared in TRAIL-resistant human leukemia cell lines (THP-1 and Molt-4) and in mildly resistant and sensitive human lymphoma/leukemia cell lines (U-937, Jurkat, and HL60) using a 3-(4,5-dimethylthiazol-2-yl)-2,5-diphenyltetrazolium bromide (MTT) assay (Figure 3A,B). TRAIL induced cytotoxicity in the three TRAIL-sensitive cell lines at 48 h, but not in the two TRAIL-resistant cell lines at concentrations ranging from 31.3 to 1000 ng/mL. In contrast, DR4-4 Fab exerted a dose-dependent cytotoxicity in all cell lines at concentrations ranging from 50 to 150 μg/mL. After 48 h of treatment with 50 µg/mL DR4-4, approximately 40% to 50% of Molt-4 and THP-1 cells were killed. At 150 µg/mL DR4-4 Fab, cell death was induced in nearly 100% of Molt-4 and approximately 75% of THP-1 cells. In normal human fibroblast cells (CCD-986SK), minimal toxicity was observed at 48 h in those treated with DR4-4 Fab (25, 50, and 100 µg/mL) and TRAIL (31.3, 62.5, and 125 ng/mL) as assessed by the MTT assay (Figure 3C). Minimal cytotoxicity of TRAIL and DR4-4 Fab in normal human fibroblast cells was also evident in morphological analysis by phase contrast microscopic analysis (Figure 3D). These results indicate that agonistic DR4-4 Fab may overcome cancer cell TRAIL resistance without significant toxicity to normal cells. 

### 2.3. DR4-4 Fab Induces Caspase-Independent Cell Death

**Figure 4:**
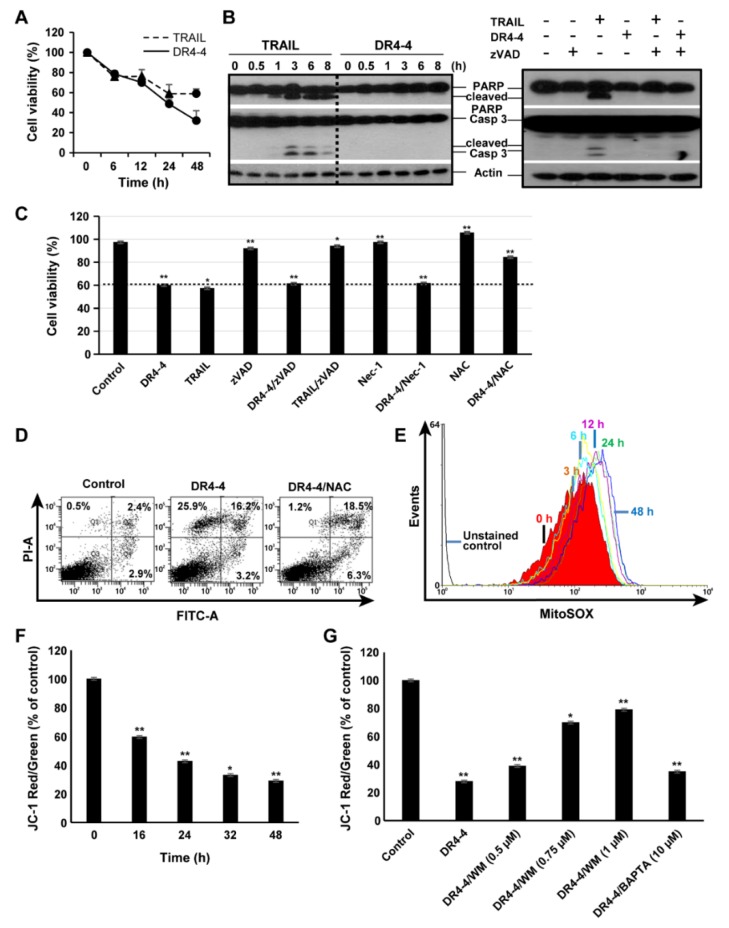
Caspase-independent and mitochondrial-induced cytotoxicity of DR4-4 Fab. (**A**) Jurkat cells (2.5 × 10^4^) were treated with TRAIL (125 ng/mL) or DR4-4 Fab (100 µg/mL) for the indicated time and cell viability was measured by MTT assay. (**B**) Cells (1 × 10^6^) were treated with TRAIL (125 ng/mL) or DR4-4 Fab (100 µg/mL) for the indicated time with or without a 3 h pretreatment with z-VAD (20 µM). Caspase-3, cleaved caspase-3, poly(ADP-ribose) polymerase (PARP), and cleaved PARP were detected by immunoblotting with specific antibodies. β-actin was used as a control to quantify the relative amount of sample loaded in each well. (**C**) MTT assay after treatment of cells with DR4-4 Fab (30 min pretreatment with or without z-VAD (20 µM), Nec-1 (50 µM), and *N*-acetyl-l-cysteine (NAC; 5 mM)) or TRAIL (30 min pretreatment with or without z-VAD (20 µM)). (**D**) Cells were treated with DR4-4 Fab (100 µg/mL) for 6 h in the presence or absence of NAC (5 mM), stained with annexin V-FITC/PI, and analyzed by flow cytometry (1 × 10^6^ cells were counted). (**E**) Mitochondrial reactive oxygen species generation was measured by flow cytometry after treatment with DR4-4 Fab (100 µg/mL) for the indicated time (0–48 h) and 5 µM MitoSOX staining. (**F**) Cells were treated with 100 µg/mL DR4-4 Fab (0, 16, 24, 32, and 48 h) and stained with JC-1 to analyze mitochondrial membrane potential (MMP). The ratio of red to green forms of JC-1 was measured by flow cytometry. (**G**) Cells were treated with 100 µg/mL DR4-4 Fab for 48 h with or without wortmannin (WM) and 1,2-bis(*o*-aminophenoxy)ethane-*N*,*N*,*N*′,*N*′-tetraacetic acid (BAPTA; 10 µM). After staining with JC-1, cells were analyzed by flow cytometry for MMP. Data presented as mean ± standard deviation (* *p* < 0.05, ** *p* < 0.005, versus control).

To investigate the mechanism of DR4-4 Fab–induced cell death, we tested the time dependency of cytotoxicity induced by the Fab fragment and TRAIL in Jurkat cells. DR4-4 Fab (100 µg/mL) and TRAIL (125 ng/mL) induced cell death in a time-dependent manner over 48 h (Figure 4A). The activation of caspase-3 and cleavage of the caspase-3 substrate, poly(ADP-ribose) polymerase (PARP), were analyzed (Figure 4B). TRAIL-induced cleavage of caspase-3 and PARP began at 1 h and continued until 8 h, whereas DR4-4 Fab did not induce cleavage over the same period. The cleavage of caspase-3 and PARP induced by TRAIL was completely blocked by pretreatment with the pan-caspase inhibitor, zVAD-fmk (zVAD). The inhibition of TRAIL-induced cell death by zVAD was also demonstrated in the MTT assay (Figure 4C). We also analyzed effects of an inhibitor of necrosis (Nec-1) and an inhibitor of ROS generation (*N*-acetyl-l-cysteine (NAC)) on DR4-4 Fab–induced cell death in the MTT assay (Figure 4C). Nec-1 only partially inhibited (10%) total cell death induced by DR4-4 Fab alone, and this rate was similar to that observed by fluorescence-activated cell sorting (FACS) analysis (data not shown). NAC decreased the level of total cell death to 50% of that induced by DR4-4 Fab alone. These results suggest that DR4-4 Fab induces cell death via a caspase-independent mechanism involving ROS generation. 

### 2.4. Mitochondria Are Involved in DR4-4 Fab–Induced Death

To investigate the types of cell death induced by DR4-4 Fab, DR4-4 Fab–treated cells were subjected to annexin V/propidium iodide (PI) staining and FACS analysis. After 6 h of DR4-4 Fab treatment, increases in apoptotic cells (Annexin V^+^/PI^−^) were not observed, while necrosis-like cell death (Annexin V^−^/PI^+^) was increased (Figure 4D), suggesting that necrosis-like cell death, and not apoptosis, was promoted by DR4-4 Fab. Furthermore, pretreatment with NAC attenuated DR4-4-induced cell death by approximately 45%, in agreement with the results observed in the MTT assay (Figure 4C). 

To confirm the involvement of mitochondrial ROS and mitochondrial disruption in the DR4-4 Fab–induced cell death, we measured the amount of mitochondrial ROS after DR4-4 Fab treatment by MitoSOX staining and flow cytometry analysis in Jurkat cells. Mitochondrial ROS generation was increased twofold after 3 h and the signal continued to increase in a time-dependent manner for 48 h (Figure 4E). 

A feature of the early stage of cell death is the damage of active mitochondria, resulting in changes in MMP and alterations to the oxidation–reduction potential of the mitochondria. JC-1 dye was used to monitor mitochondrial health and MMP. Mitochondrial damage and depolarization are indicated by a decrease in the red to green ratio of fluorescence intensity (aggregates:monomers) of JC-1 dye. This ratio was gradually decreased over 48 h in DR4-4 Fab–treated cells, indicating mitochondrial disruption during DR4-4 Fab–induced cell death (Figure 4F). 

**Figure 5:**
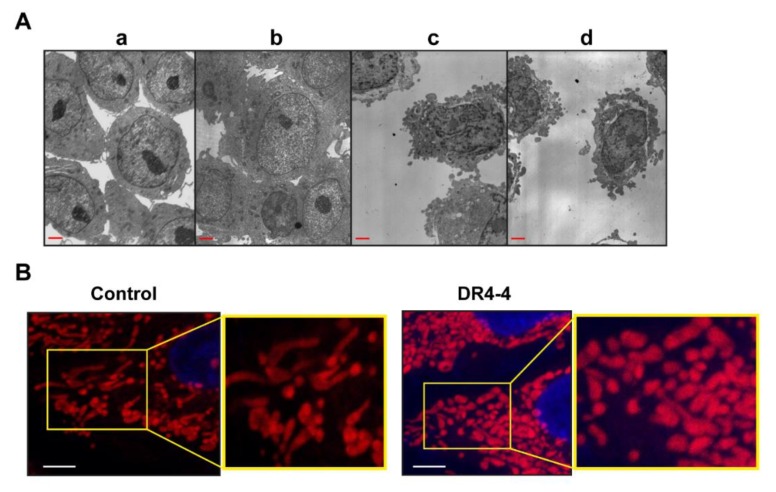
Morphological observation of cells treated with DR4-4 Fab. (**A**) Representative transmission electron microscopy images taken after THP-1 cell treatment with 50 µg/mL DR4-4 Fab for 0 h (**a**), 24 h (**b**), or 48 h (**c**,**d**). Scale bar, 2.5 µm. (**B**) Representative images showing morphological changes in the mitochondrial network of HeLa cells treated with 50 µg/mL DR4-4 Fab for 24 h observed by confocal microscopy (100×) after MitoTracker Red FM staining of mitochondria. Scale bar, 5 µm. The parts of the images were enlarged and presented in yellow squares.

Cellular morphology was investigated using transmission electron microscopy (TEM) revealing characteristics of necrosis-like death (loss of plasma membrane integrity, swollen cells and mitochondria) rather than apoptotic morphology (cellular shrinkage, nuclear fragmentation) in a human monocyte leukemia cell line (THP-1) (Figure 5A). For unknown reasons, image analysis of Jurkat cells by TEM or confocal microscopy was not optimal. Morphological changes in the mitochondrial network of HeLa cells (human cervical cancer cell line) treated with DR4-4 Fab for 24 h were observed using confocal microscopy (Figure 5B). Compared to the elongated and fused forms of mitochondrial networks in untreated control cells, fragmented forms were dominant in DR4-4 Fab–treated cells, indicating the involvement of changes in mitochondrial dynamics in DR4-4 Fab–induced cell death. It is suggested that the combination of these morphological changes and damage resulting from the production of mitochondrial ROS contribute to DR4-4 Fab–induced necrosis-like cell death; however, other mechanisms may also contribute to this response.

### 2.5. DR4-4 Fab Activates p38 MAPK, Inhibits c-FLIP, and Increases Cell Death Rates in Combination with TRAIL or γ-Irradiation

**Figure 6:**
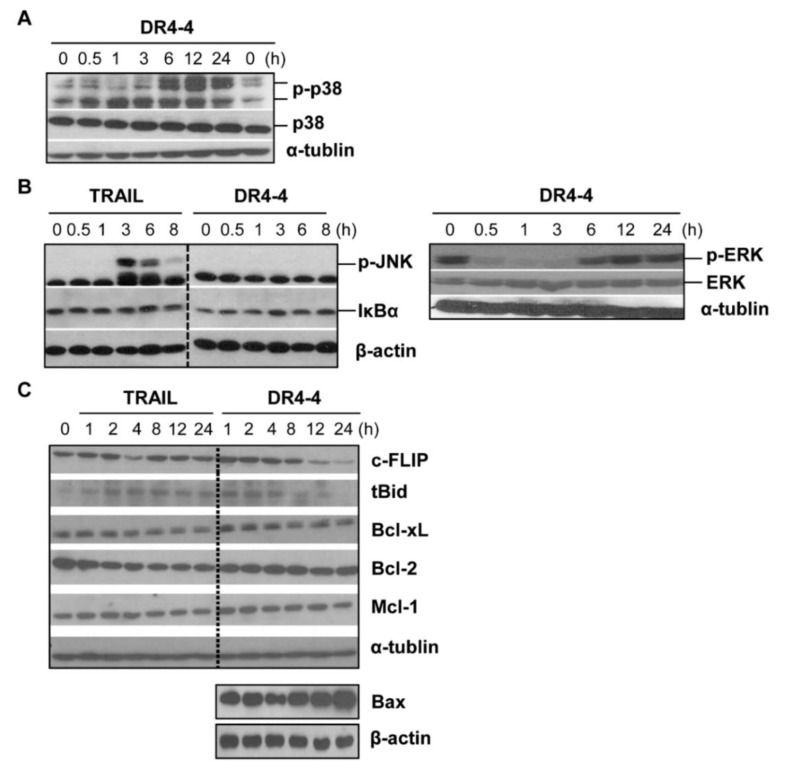
Immunoblot analysis of signaling molecules induced by DR4-4 Fab. Immunoblotting performed after the indicated length of treatment of Jurkat cells (1 × 10^6^) with TRAIL (125 ng/mL) or DR4-4 Fab (100 µg/mL). (A) P-p38 and p38 were detected with specific antibodies. (B) P-JNK and IκBα were detected after the treatment of TRAIL or DR4-4 Fab. P-ERK and ERK were analyzed only after the treatment with DR4-4 Fab. (C) C-FLIP, tBID, Bcl-xL, Bcl-2, Mcl-1, and Bax were analyzed with specific antibodies after the treatment of TRAIL or DR4-4 Fab. α-tubulin and β-actin were used to quantify the relative amount of sample loaded into each well. Bax, Bcl-2-associated X protein; Bcl-2, B-cell lymphoma 2; Bcl-xL, B-cell lymphoma extra-large; c-FLIP, cellular FADD-like IL-1β-converting enzyme–inhibitory protein; ERK, extracellular signal-regulated kinase; IκBα, nuclear factor κB inhibitor α; JNK, c-Jun N-terminal kinase; Mcl-1, myeloid cell leukemia 1; p-, phosphorylated protein; t-Bid, truncated cytosolic BH3 interacting-domain death agonist.

We used immunoblotting to examine whether MAPKs (p38, c-Jun N-terminal kinase (JNK), and extracellular signal-regulated kinase (ERK)) and NF-κB were involved in DR4-4 Fab–induced cell death in Jurkat cells. DR4-4 Fab activated p38, but not JNK, ERK, or NF-κB in Jurkat cells (Figure 6A,B). Activation of p38 MAPK was evident after 6 h and continued until 24 h after DR4-4 Fab treatment. Activation of JNK and NF-κB was not detected in the 8 h following treatment with DR4-4 Fab. Activation of JNK by treatment with TRAIL, however, was clearly observed at 3 h (Figure 6B). NF-κB was not activated by TRAIL, an observation that was consistent with several reports in various cancer cell types [3,34,35,36]. The level of phosphorylated ERK was clearly decreased by DR4-4 Fab treatment in the short term (≤3 h) but recovered at later timepoints (≥6 h) for unknown reasons. It is suggested that the activation of p38 is involved in Jurkat cell death induced by DR4-4 Fab. The activation of p38 in DR4-4 Fab–induced cell death was also indicated by the change in MMP in cells pretreated with an inhibitor of MAPK, wortmannin (WM) (Figure 4G). Various concentrations of WM (0.5, 0.75, and 1 µM) inhibited the decrease in the ratio of aggregate to monomer forms of JC-1 dye induced by DR4-4 Fab in a dose-dependent manner, while an intracellular calcium chelator, 1,2-bis(*o*-aminophenoxy)ethane-*N*,*N*,*N*′,*N*′-tetraacetic acid (BAPTA) (10 µM), did not. 

c-FLIP is a major antiapoptotic protein that is initiated by the interaction of TRAIL agonists or stress signals with DR4 [37]. At 100 µg/mL, DR4-4 Fab decreased the level of c-FLIP at 12 and 24 h (Figure 6C), suggesting a possible role for c-FLIP in DR4-4 Fab–induced cell death. The mechanism by which the reduced level of c-FLIP affects cell death without caspase activation is not clear. 

TNFα, Fas, TRAIL, or TRAIL agonists can activate caspase-8 to truncate cytosolic BH3 interacting-domain death agonist (Bid), a pro-apoptotic protein, to truncated Bid (tBid) during cell death [38]. Upon translocation to the mitochondria, tBid contributes to cytochrome C release; however, in DR4-4 Fab–treated Jurkat cells, only a moderate increase in tBid was observed at very early sampling times in comparison to untreated cells (Figure 6C). It is possible that the level of Bid cleavage may not have been high due to low levels of caspase activity. DR4-4 tended to increase the level of Bcl-2-associated X protein (Bax) at 24 h which is associated with the mitochondrial pathway. Protein levels of B-cell lymphoma extra-large (Bcl-xL), B-cell lymphoma 2 (Bcl-2), and myeloid cell leukemia 1 (Mcl-1) were not significantly changed by DR4-4 Fab treatment over 24 h.

**Figure 7:**
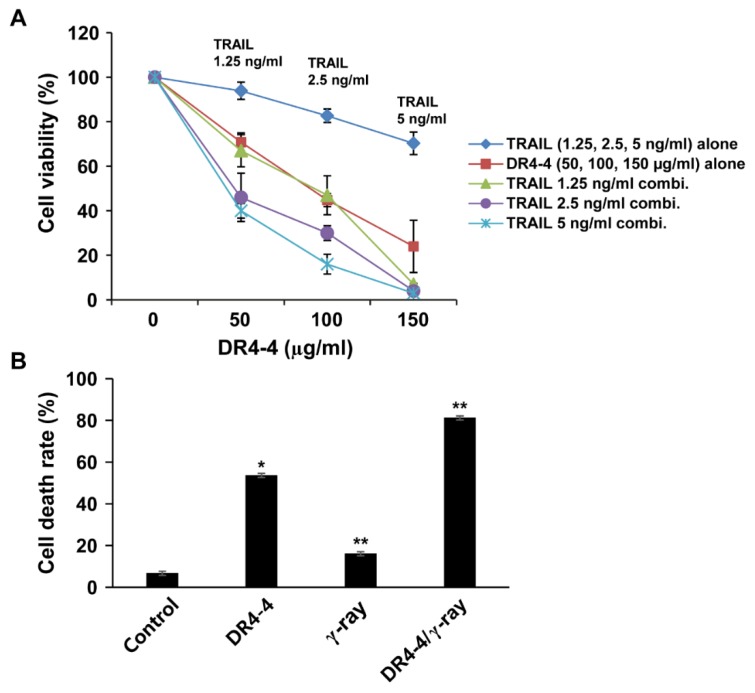
Enhanced rates of cell death following cotreatment with DR4-4 Fab and TRAIL or γ-irradiation. (**A**) Cytotoxicity of cotreatment with DR4-4 Fab and TRAIL as measured by MTT assay. Cell viability for single treatment with TRAIL is also presented with concentrations indicated (upmost line). (**B**) Cells pre-irradiated with γ-radiation (absorbed dose = 2 Gy) were treated with DR4-4 Fab (100 µg/mL) for 24 h. Cell death was measured by MTT assay. Data presented as mean ± standard deviation (* *p* < 0.05, ** *p* < 0.005, versus control).

Since DR4-4 Fab exerts cytotoxicity by mechanisms different from that of TRAIL, we tested whether DR4-4 Fab could sensitize Jurkat cells to TRAIL-induced cell death. Both individual and combined treatments with DR4-4 Fab and TRAIL are presented in Figure 7A. Treatment with DR4-4 Fab at 50, 100, and 150 µg/mL resulted in cell viabilities of approximately 70%, 45%, and 25%, respectively. Pretreatment of cells with TRAIL (1.25, 2.5, or 5 ng/mL) followed by DR4-4 Fab treatment (50, 100, or 150 µg/mL) revealed that the combinations involving 2.5 or 5 ng/mL TRAIL resulted in remarkable increases in cytotoxicity. Cells pretreated with 5 ng/mL TRAIL (single-treatment viability = 70%) followed by treatment with 100 µg/mL DR4-4 Fab (single-treatment viability = 45%) resulted in a viability of 15%. This additive effect suggested that these agents were functioning independently, perhaps reflecting differences in cell sensitivity to each agent. As expected, based on the single-treatment viability of each agent, the combination of 150 µg/mL DR4-4 Fab with 5 ng/mL TRAIL pretreatment induced near-complete cell death. The effectiveness of DR4-4 Fab was also tested in combination with γ-irradiation (Figure 7B). The rate of cell death following a single treatment with DR4-4 Fab (100 µg/mL) for 24 h was 54%. The death rate induced by a single γ-radiation (2 Gy) treatment (15%) was increased to a total of 80% when this was followed by treatment with DR4-4 Fab (100 µg/mL) for 24 h.

## 3. Discussion

Agonistic TRAIL receptor mAbs or Fab fragments such as DR4-4 Fab and other mAbs [5,6,8,9,12] may provide more efficient immunotherapy for cancer than TRAIL. Many DR agonists have been demonstrated to be safe and promising for cancer immunotherapy in phase I, II, and III clinical trials [28,31,32]. 

In this study, we demonstrated the toxic activity of a novel agonistic anti-DR4 Fab in TRAIL-resistant and mildly TRAIL-resistant cells, including leukemia and lymphoma cell lines (Jurkat, THP-1, Molt-4, and U937), as well as TRAIL-sensitive leukemia cells (HL60), but not in normal human cells. The results indicate that DR4-4 Fab can overcome TRAIL resistance in some cancer cells. The mechanism of cell death triggered by DR4-4 Fab in the Jurkat cell line, which is composed of cells with differing TRAIL sensitivities and a high proportion of highly resistant cells [3], was different from the mechanisms involved in TRAIL-induced cell death. DR4-4 Fab does not induce caspase activation, PARP cleavage, cellular shrinkage, or annexin V^+^/PI^–^ staining of the cellular membrane, indicating the induction of non-apoptotic, caspase-independent cell death. Another agonistic mAb (AD5-10) activating a caspase-independent signaling pathway via DR5 in Jurkat cells has previously been reported [23]. DR4 appears to have an ability to trigger nonclassical mechanisms of cell death by interacting with specific agonistic Abs, different from the well-known caspase-dependent role of DR4. Because DR4-4 Fab and TRAIL use distinct mechanisms to kill cancer cells, their combination can lead to increased cytotoxic effects. We demonstrated that both TRAIL and γ-irradiation in combination with DR4-4 Fab treatment resulted in further decreases in Jurkat cell viability when compared to DR4-4 Fab treatment alone. 

The mechanisms involved in DR4-4 Fab–induced cell death were found to involve changes in a variety of signal transduction molecules and cellular events, including p38 MAPK, c-FLIP, Bax, mitochondrial ROS, and MMP. Impaired mitochondria produce ROS, which leads to cellular damage and various types of cell death [39,40]. Increased mitochondrial ROS and decreased MMP were observed in Jurkat cells treated with DR4-4 Fab. Morphological changes in the mitochondrial network from fused and elongated to fragmented forms were observed after DR4-4 Fab treatment, supporting the involvement of mitochondria in DR4-4 Fab–induced cell death. Phosphorylated p38 can reduce MMP and increase ROS production, thereby inducing the mitochondrial pathway of cell death [41]. Free radicals can also activate MAPKs in a positive feedback loop, further contributing to cellular damage [42,43]. Phosphorylation of p38 MAPK also regulates the degradation of c-FLIP [44]. Therefore, an overall pathway that includes p38 MAPK activation, c-FLIP degradation, MMP reduction, and ROS production may explain DR4-4 Fab–induced cell death in Jurkat cells. Previous studies have demonstrated the involvement of mitochondria in the death of human colon cancer cells treated with TRAIL and an agonistic anti-DR4 mAb [45]. Further studies are required to clearly establish the link between signaling molecules and cell death induced by DR4-4 Fab in cancer cells. 

An increasing rate of therapeutic resistance has been identified as a problem in the treatment of leukemia [46]. TRAIL resistance in some cancer cells involves abnormal function of the death-inducing signaling complex (DISC) due to inactivation or downregulation of caspase-8 [47] or inhibition of DISC by c-FLIP [48]. Therefore, the ability of DR4-4 Fab to induce cell death without caspase activation and to decrease levels of c-FLIP may provide an alternative treatment option to circumvent TRAIL resistance in cancer therapy. 

Other agonistic mAbs to DR4 and DR5 were generally found to induce cancer cell death through apoptosis in previous studies [23,28,29,30,31]; however, autophagy has also been implicated in their effects [23]. We suggest that DR4-4 Fab–induced cell death involves necrosis-like death based on the observed morphology by TEM, PI-single positive staining in FACS, and partial inhibition of cell death by a necrosis inhibitor (Nec-1). The partial inhibition of cell death by the necrosis inhibitor suggests the involvement of other mechanisms of cell death. Additionally, autophagic inhibitors (3-methyladenine and bafilomycin-1) did not block DR4-4 Fab–induced cell death (data not shown). 

Necroptosis-based therapeutic strategies have been emphasized for bypassing drug resistance in leukemia [46]. Further exploration of the properties of DR4-4 Fab responsible for triggering necrosis or necrosis-like death would be invaluable for developing agents to overcome TRAIL resistance and treat various cancer cells including leukemia cells. DR4-4 Fab may not only be a good candidate as a single therapeutic for the treatment of TRAIL-resistant cancer cells, but also as a promising agent for combination therapy with TRAIL or γ-irradiation, which may also allow for a lower required dosage of DR4-4 Fab. In all experiments, DR4-4 Fab was effective at higher doses than TRAIL. Converting DR4-4 Fab into the immunoglobulin G (IgG) form of DR4-4 might also be helpful for lowering the required dosage and increasing the efficacy of the Ab in vivo.

In summary, we have characterized the basic and critical properties of an agonistic recombinant human monovalent mAb Fab, DR4-4, against human DR4. Cell death induced by DR4-4 Fab is caspase-independent necrosis or necrosis-like death and involves p38 MAPK, mitochondria, ROS, and c-FLIP. 

## 4. Materials and Methods

### 4.1. Reagents

Nec-1, NAC, WM, and BAPTA were purchased from Merck (Darmstadt, Germany). Annexin V-FITC/PI and JC-1 dye were from Abcam (Cambridge, UK). MitoSOX and MitoTracker Red FM were from Thermo Fisher (Waltham, MA, USA). Antibodies against PARP, caspase-3, β-actin, α-tubulin, p38, p-JNK, p-ERK, IκBα, c-FLIP, tBid, Bcl-xL, Bcl-2, Bax, and Mcl-1 were obtained from Cell Signaling Technology (Danvers, MA, USA). Horseradish peroxidase (HRP)-conjugated mouse anti–human IgG (Fab specific) Ab and MTT were purchased from Sigma-Aldrich (St. Louis, MO, USA). The z-VAD was purchased from Santa Cruz Biotechnology (Dallas, TX, USA) and dissolved in dimethyl sulfoxide. sTRAIL and DR4/DR5 were purchased from Koma Biotech (Seoul, Korea) and Strathmann Biotec (Hamburg, Germany), respectively. Human DcR1/Fc chimera and human DcR2/Fc chimera were from R&D Systems (Minneapolis, MN, USA).

### 4.2. Phage Display and Purification of Soluble Fab

Detailed methods for phage display and purification of soluble Fab were described previously [49]. For panning, the wells of an ELISA plate were coated with 7 µg/mL recombinant human DR4 in phosphate-buffered saline (PBS; pH 7.4). Sequence analysis was performed using the National Center for Biotechnology Information IgBLAST program (https://www.ncbi.nlm.nih.gov/projects/igblast/).

Protein expression of DR4-4 Fab containing (His)_5_ tag was induced in *Escherichia coli* and purified using an ÄKTAprime plus instrument with a HisTRAP column (GE Healthcare, Uppsala, Sweden). The purified Fab was visualized through immunoblotting using anti–human IgG (Fab specific) Ab and Coomassie blue staining.

### 4.3. Cell Culture

All cell lines except CCD-986SK (normal human fibroblast) were purchased from the American Type Culture Collection (Manassas, VA, USA). THP-1 (human monocytic leukemia), Molt-4 (human T-cell lymphoblastic leukemia), U937 (human lymphoma), Jurkat (human T cell leukemia), CCD-986SK, and HL60 (human myeloid leukemia) cell lines were cultured in Roswell Park Memorial Institute (RPMI) 1640 medium supplemented with 10% heat-inactivated fetal bovine serum (FBS), 2 mM glutamine, and 100 µg/mL penicillin/streptomycin. CCD-986SK cells were kindly provided by S.-Y. Jeong (Ajou Univ.). For Molt-4 cells, FBS content was increased to 20%. For HeLa (human cervical adenocarcinoma), the cell line was cultured in Dulbecco’s Modified Eagle Medium (DMEM) with same supplements as RPMI. 

### 4.4. Immunoblotting

For the analysis of the signal molecules, cells were cultured in a 12-well plate and incubated with Fab or TRAIL in the presence or absence of an inhibitor. Cells were lysed using a RadioImmunoPrecipitation Assay (RIPA) lysis buffer. Samples were collected, 5× sample buffer was added to each sample, and the samples were heated to 95 °C for 5 min. Samples were separated by 10% sodium dodecyl sulfate-polyacrylamide gel electrophoresis and proteins were blotted on nitrocellulose paper. Specific Abs against signal molecules were used according to manufacturer instructions. Enhanced chemiluminescence (ECL) reagent (Amersham Biosciences, Piscataway, NJ, USA) was applied to the membrane for band visualization.

### 4.5. Direct Binding and Competitive ELISA

The ELISA was performed as previously described [50]. Antigens coated onto the wells were human DR4 (Strathmann Biotec #9525245), human DR5 (Strathmann Biotec #9525251), human DcR1/Fc chimera (R&D Systems #630-TR/CF), and human DcR2/Fc chimera (R&D Systems #633-TR). The human DcR1 peptide (Abcam #ab6096, 13 amino acids) and DcR2 peptide (Abcam #ab8379, 15 amino acids) were also used. The bound Fabs were detected with HRP-conjugated anti–human IgG (Fab specific) Ab.

### 4.6. Affinity Analysis by SPR

SPR analysis was performed using a Biacore 2000 instrument (Biacore AB, Uppsala, Sweden) and BIAevaluation software to measure affinity of DR4-4 Fab for DRs and decoy receptors. A nitrile triacetic acid (NTA) sensor chip (GE Healthcare, Uppsala, Sweden) was used for Fab immobilization. 

### 4.7. FACS Analysis for Cellular Binding of DR4-4 Fab

Cells were pelleted by centrifugation for 3 min at 800× *g*, resuspended in RPMI 1640 medium, and incubated with DR4-4 Fab for 1 h at 4 °C. Unbound domain protein was removed by three 15 min washes in PBS at room temperature, and the cells were fixed with 4% paraformaldehyde. The cells were incubated with rabbit IgG (2 µg/mL) for 1 h and then with FITC-conjugated goat anti–rabbit IgG (1 µg/mL) for 1 h. The FITC signal was analyzed by flow cytometry using a FACSAria III (Becton Dickinson, Franklin Lakes, NJ, USA) and FlowJo-7 software. 

### 4.8. Cytotoxicity Test

Cytotoxicity or survival after treatment of cells with Abs was measured using an MTT assay as described previously [3]. 

### 4.9. Mitochondrial ROS and MMP Measurement

For mitochondrial ROS and MMP measurement, cells were treated with DR4-4 Fab, stained with MitoSOX (for ROS) and JC-1 (for MMP) dyes following each manufacturer’s protocols and analyzed by flow cytometry.

### 4.10. Confocal Microscopy for Mitochondrial Morphology

Cells were grown to confluence in 12-well plates containing 18 mm cover slips. Cells were incubated with 50 µg/mL DR4-4 Fab for 24 h at 37 °C, washed in ice-cold PBS, and fixed with 4% paraformaldehyde. Mitochondria were stained with MitoTracker, nuclei were stained with Hoechst 33342 (Invitrogen, Carlsbad, CA, USA), and the specimens were mounted using mounting medium. Images were acquired at 100× using a Zeiss LSM 710 confocal microscope (Carl Zeiss, Oberkochen, Germany). 

### 4.11. Electron Microscopy for Cellular Image Analysis

Cells were washed six times with 0.1 M sodium cacodylate buffer, and treated with 1% OsO_4_/1% potassium ferrocynanide/0.1 M sodium cacodylate buffer for 30 min. Cells were washed six times and dehydrated in gradually increasing concentrations of ethanol (30–100%). Cells were embedded in resin and photographed using a transmission electron microscope (Zeiss EM 902A; Carl Zeiss, Oberkochen, Germany).

### 4.12. γ-Irradiation

Cells were irradiated at room temperature with a laboratory γ-irradiator (Atomic Energy of Canada Ltd., Mississauga, ON, Canada) at a dose rate of 3.81 Gy/min. 

### 4.13. Statistical Analysis

All results are presented as mean ± standard deviation. The *t*-test was employed for statistical analyses of data from two to five independent experiments. The significance of differences from control groups are shown as values of probability and indicated with asterisks: * *p* < 0.05 and ** *p* < 0.005. 

## Abbreviations

Ab	antibody
BAPTA	1,2-bis(*o*-aminophenoxy)ethane-*N*,*N*,*N*′,*N*′-tetraacetic acid
Bcl-2	B-cell lymphoma 2
Bcl-xL	B-cell lymphoma extra-large
Bid	BH3 interacting-domain death agonist
c-FLIP	cellular FADD-like IL-1β-converting enzyme–inhibitory protein
DcR	decoy receptor
DISC	death-inducing signaling complex
DR	death receptor
ELISA	enzyme-linked immunosorbent assay
ERK	extracellular signal-regulated kinase
Fab	antigen binding fragment
FACS	fluorescence-activated cell sorting
FITC	fluorescein isothiocyanate
IκBα	nuclear factor κB inhibitor α
JNK	c-Jun N-terminal kinase
mAb	monoclonal antibody
Mcl-1	myeloid cell leukemia 1
MMP	mitochondrial membrane potential
MTT	3-(4,5-dimethylthiazol-2-yl)-2,5-diphenyltetrazolium bromide
NAC	*N*-acetyl-l-cysteine
NF-κB	nuclear factor κB
p38 MAPK	p38 mitogen-activated protein kinase
PBS	phosphate-buffered saline
ROS	reactive oxygen species
SPR	surface plasmon resonance
sTRAIL	soluble TRAIL
tBid	truncated Bid
TNF	tumor necrosis factor
TRAIL	tumor necrosis factor–related apoptosis-inducing ligand
V_H_	heavy chain
V_L_	light chain
WM	wortmannin
